# Objectively-Measured Physical Activity and Sedentary Time are Differentially Related to Dietary Fat and Carbohydrate Intake in Children

**DOI:** 10.3389/fpubh.2018.00198

**Published:** 2018-07-20

**Authors:** Genevieve F. Dunton, Sydney G. O'Connor, Britni R. Belcher, Jaclyn P. Maher, Susan M. Schembre

**Affiliations:** ^1^Preventive Medicine, University of Southern California, Los Angeles, CA, United States; ^2^Department of Kinesiology, University of North Carolina, Greensboro, NC, United States; ^3^University of Texas MD Anderson Cancer Center, Houston, TX, United States

**Keywords:** physical activity, sedentary behavior, dietary intake, accelerometer, 24 h dietary recall, children

## Abstract

**Background:** Research on the clustering of physical activity, sedentary, and dietary intake behaviors in children has relied on retrospective and parent-report measures, which may obscure true associations. The current study combined objectively-measured moderate-to-vigorous intensity physical activity (MVPA) and sedentary time (ST) data from waist-worn accelerometers, with multiple child-report 24-h dietary recalls to assess specific components of dietary intake (i.e., dietary fat, carbohydrates, protein; glycemic load, fruits and vegetables) in children.

**Methods:** Participants (*n* = 136, ages 8–12 years) wore an accelerometer for 7 days. On two of those days, children completed 24-h recall phone interviews to assess dietary intake.

**Results:** After adjusting for child age, sex, ethnicity, annual household income, and body mass index (BMI) percentile; ST was positively associated with percent dietary fat intake, and negatively associated with percent dietary carbohydrate intake and glycemic load (*p*'s < 0.01). MVPA was positively associated with percent dietary carbohydrate intake and daily glycemic load, and negatively associated with percent dietary fat intake (*p*'s < 0.05).

**Conclusion:** Despite its direct health benefits, physical activity may be associated with consuming greater proportion of total intake from carbohydrates, especially those with a higher glycemic index. Further research is needed to understand the differential implications of these unique behavioral interrelations for diabetes, cardiovascular, and obesity risk.

## Introduction

Low levels of physical activity, high levels of sedentary behavior, and poor dietary intake (e.g., high dietary fat, high glycemic load) in childhood are modifiable health behaviors that have been implicated in elevated lifelong risk of obesity, diabetes, and cardiovascular disease (1–7). Understanding the interrelationships among physical activity, sedentary behavior, and dietary intake in children is relevant to the design and development of health promotion programs. Interventions that are able to successfully bring about change in more than one health behavior (i.e., multiple behavior change) may be more cost-effective than those that only impact single behaviors (8). However, physical activity and sedentary behaviors may be associated with specific aspects of dietary intake in multifaceted ways that are not well understood, particularly in youth (9).

Studies examining the relationships of physical activity and sedentary behavior with diet in children and adolescents suffer from limitations that circumscribe their ability to inform interventions. First, studies on this topic tend to use retrospective and parent-report measures of usual physical activity behavior (9), which are subject to recall and reporting biases, particularly in children (10). A second limitation of these studies is the failure to distinguish between aspects of dietary intake (e.g., fat, carbohydrates, protein, glycemic load, fruits, and vegetables) that may be differentially associated with specific energy expenditure behaviors (9).

The current study aimed to address these limitations by combining objectively-measured moderate-to-vigorous intensity physical activity (MVPA) and sedentary time (ST) using a waist-worn accelerometer with multiple child-report 24-h dietary recalls to assess specific components of dietary intake in children. The primary objective was to determine whether MVPA and ST are differentially related to specific components of dietary intake (i.e., fat, carbohydrates, protein, daily glycemic load, fruits and vegetables) in children.

## Materials and methods

### Overview

The current cross-sectional analyses used baseline data from the Mothers' and Their Children's Health (MATCH) study (11), which is a longitudinal investigation of parenting factors and obesity in a sample of mothers and children. This study was approved by the Institutional Review Board at the University of Southern California. Parents provided written consent/permission for children to participate, and children provided written assent.

### Participants

Participants included a convenience sample of ethnically-diverse 8–12 year-old children recruited from demographically-representative public elementary schools and after-school programs in the greater Los Angeles metropolitan area. Children were recruited through informational flyers and in-person recruitment events. Inclusion criteria were: (1) child is in the 3rd−6th grade, (2) ≥50% of child's custody resides with the mother, and (3) child is able to read English. Exclusion criteria were: (1) currently taking medications for thyroid function or psychological conditions such as depression, anxiety, mood disorders, and ADHD, (2) health issues that limit physical activity, (3) enrolled in special education programs, (4) currently using oral or inhalant corticosteroids for asthma, (5) mother is pregnant, (6) child classified as underweight by a BMI percentile <5% adjusted for sex and age, and (7) mother works more than two weekday evenings (between the hours of 5–9 p.m.) per week or more than 8 h on any weekend day.

### Procedures

During the 7-day data collection period, children wore an accelerometer during waking hours and completed two parent-assisted 24-h dietary recalls by phone with trained interviewers. This study was carried out in accordance with the recommendations of the Human Subjects Institutional Review Board at the University of Southern California. The protocol was approved by the Health Sciences Review Board. All subjects gave written informed consent/assent in accordance with the Declaration of Helsinki. All study protocols, descriptions of analytic methods, materials, and data are available upon request to the authors.

### Measures

#### Accelerometer

The Actigraph, Inc. GT3X model accelerometer was used for measurement of MVPA and ST. The device was worn on the right hip, attached to an adjustable belt, at all times except sleeping, bathing, or swimming. The device was set to collect movement data in activity count units for each 30-s epoch. Meterplus software (Santech, San Diego, CA) was used to identify periods of non-wear (>60 continuous minutes of zero activity counts) and valid days (at least 10 h of wear). Cut-points for time spent in MVPA and ST were consistent with studies of national surveillance data (12, 13) using age-specific thresholds for children generated from the Freedson prediction equation equivalent to 4 METs (13–18). ST was defined as activity < 100 counts per minute (19, 20).

#### Dietary assessment

Usual dietary intake was assessed using two 24-h dietary recalls. Each recall was conducted over the phone by trained research staff using a five-pass interview approach (21, 22). The 24-h dietary recalls were collected from children with assistance from mothers. Children were asked to recall what they ate during the course of the prior day (midnight to midnight). It was preferred that the recalls consisted of one weekday and one weekend day. However, if a participant missed one or both scheduled calls, make-up calls were made within the remaining days of the data collection period that may have led to the inclusion of two weekdays or two weekend days in the recall. Dietary intake data were analyzed using Nutrition Data System for Research software versions 2013 and 2015. Usual daily dietary intake was calculated by averaging dietary intake across recall days. Dietary intake amounts for fat, carbohydrate, and protein were reported as a percent of total energy intake (e.g., percent of calories from dietary fat) to account for plausible differences in energy requirements across the study sample (23, 24). Dietary intake for fruit and vegetables was calculated by dividing the reported servings by total kcal of energy consumed. This amount was then multiplied by 1,000 kcals to determine adjusted intake per 1,000 kilocalories consumed. Dietary outcome variables were as follows: percent energy from dietary fats, carbohydrates, and protein; glycemic load; and fruits and vegetables servings per 1,000 kcal. A food's glycemic load is determined by multiplying its glycemic index (i.e., relative ranking of a carbohydrate according to how it quickly it is digested, absorbed in the blood, and metabolized) by the amount of carbohydrate (g) contained in that food and dividing by 100.

#### Anthropometric assessments

Height (m) and weight (kg) were measured in duplicate using an electronically calibrated digital scale (Tanita WB-110A) and professional stadiometer (PE-AIM-101). Body mass index (BMI; kg/m^2^) and CDC age and sex-specific BMI percentiles were determined using EpiInfo 2005, Version 3.2 (CDC, Atlanta, GA).

#### Demographic factors

Mothers completed paper questionnaires reporting on their child's ethnicity and annual household income. Children self-reported their age and sex.

### Data analyses

Fruit and vegetable intake was square root transformed to meet normal distribution assumptions in models. Bivariate analyses consisting of Pearson's correlations and *t*-tests examined the associations of covariates (i.e., child age, sex, ethnicity [Hispanic vs. non-Hispanic], annual household income [divided into quartiles: <$35,000; $35,001–$75,000; $75,001–$105,000; ≥$105,001) with the dietary outcome variables. Multiple linear regression analyses examined the associations of MVPA and ST with dietary outcomes controlling for child age, sex (male vs. female), ethnicity (Hispanic vs. non-Hispanic), annual household income (quartiles), and BMI percentile. Separate models were run to test the associations of MVPA and ST with each dietary outcome.

## Results

### Data availability

Of the 464 mother-child dyads interested in the study, 132 dyads could not be reached for eligibility screening, 22 dyads declined to be screened, 62 dyads did not meet eligibility criteria, and 46 dyads either did not attend their enrollment session or were no longer interested. A total of 202 children (and their mothers) enrolled in the study. Of this number, 14 children did not have at least two valid days of accelerometer data, an additional 46 children did not have at least 2 days of 24-h diet recall data, and an additional 6 children were missing height and weight data—leaving an analytic sample of 136 children. The analytic and excluded sample did not differ in terms of child age, sex, ethnicity, annual household income, or BMI percentile. Of the analytic sample, 10 children (7.4%) had 24-h dietary recall data for two weekend days, and 23 children (16.9%) had 24-h dietary recall data on two weekdays. Furthermore, 10 children (7.4%) had < 4 valid days (>10 h) of accelerometer data.

### Descriptive statistics and bivariate correlations

Table 1 shows demographic characteristics and descriptive statistics for the study variables. MVPA was moderately negatively associated with ST (*r* = −0.61, *p* < 0.001). Child BMI percentile was positively associated with percent energy intake from dietary fat (*r* = 0.22, *p* = 0.01), and negatively associated with percent energy intake from carbohydrates (*r* = −0.21, *p* = 0.02) and daily glycemic load (*r* = −0.27, *p* = 0.01). Hispanic children had a greater percent energy intake from protein (*M* = 16.36, *SD* = 4.53) than non-Hispanic children (*M* = 14.16, *SD* = 3.19) (*t* = 3.23, *p* = 0.002). Hispanic children also reported consuming a smaller percent energy intake from carbohydrates (*M* = 50.83, *SD* = 8.07) than non-Hispanic children (*M* = 53.62, *SD* = 7.24; *t* = −2.12, *p* = 0.04). Further, Hispanic children consumed a lower glycemic load (*M* = 118.67, *SD* = 42.65) than non-Hispanic children (*M* = 144.89, *SD* = 41.20; *t* = −3.64, *p* < 0.001). Child age, sex, and annual household income were not related to any of the dietary outcomes.

**Table 1:** Demographics and descriptive statistics for child participants (8–12 years) (*n* = 136).

**Variable**	***n* (%)**	**M (SD)**
**SEX**		
Male	65 (47.8)	
Female	71 (52.2)	
**ETHNICITY**		
Hispanic	72 (52.9)	
Non-hispanic	64 (47.1)	
**ANNUAL HOUSEHOLD INCOME**		
< $35,000	34 (25.0)	
$35,001–$74,999	45 (33.1)	
$75,000–$104,999	27 (19.9)	
$105,000 and above	30 (22.1)	
Age (years)		9.63 (0.93)
BMI percentile		64.36 (28.71)
Mean daily MVPA (min.)		58.45 (24.62)
Mean daily ST (min. per hour of wear)		34.47 (5.33)
Mean daily kcal		1,748.10 (446.62)
Mean daily dietary fat (% of total kcal)		32.51 (6.35)
Mean daily dietary carbohydrate (% of total kcal)		52.14 (7.79)
Mean daily dietary protein (% of total kcal)		15.33 (4.09)
Mean daily glycemic load		131.01 (43.83)
Mean daily fruits + veg. (servings) per 1000 kcal		1.98 (1.18)

*kcal, kilocalories; BMI, body mass index; MVPA, moderate-to-vigorous physical activity on valid days measured by waist-worn accelerometer; ST, sedentary time measured by waist-worn accelerometer. Dietary intake variable measured by multiple 24-h recalls. Dietary intake variables are not transformed*.

### Covariate-adjusted associations of MVPA and ST with dietary intake variables

Results of the multiple linear regression analyses of MVPA and ST predicting dietary intake variables controlling for child age, sex, ethnicity (Hispanic vs. not Hispanic), annual household income (quartiles), and BMI percentile are shown in Tables 2, 3, respectively. MVPA was positively associated with percent energy intake from carbohydrates and glycemic load, and negatively associated with percent energy intake from fat. ST was positively associated with percent energy intake from fat, and negatively associated with percent energy intake from carbohydrates and glycemic load.

**Table 2:** Summary of multiple linear regression analysis for moderate-to-vigorous physical activity (MVPA) predicting dietary intake variables in children (8–12 years) (*n* = 136).

	**Dietary fat (% total kcal)**	**Mean daily dietary carbohydrate (% of total kcal)**	**Mean daily dietary protein (% of total kcal)**	**Mean daily glycemic load**	**Mean daily fruits + veg. (servings) per 1,000 kcal**
**Variable**	β	β	β	β	β
Age	−0.055	0.031	0.029	0.052	−0.005
Male	0.078	−0.164	0.188^*^	−0.152	−0.089
Hispanic	0.059	−0.176^*^	0.245^**^	−0.179^*^	−0.081
Annual household income (quartiles)	0.032	−0.055	0.058	−0.040	0.007
BMI percentile	0.099	−0.155	0.136	−0.196^*^	0.119
MVPA	−0.214^*^	0.220^*^	−0.085	0.192^*^	0.016

MVPA, average daily minutes moderate-to-vigorous physical activity on valid days measured by waist-worn accelerometer. kcal, kilocalories; BMI, body mass index; Dietary intake variable measured by multiple 24-h recalls. Fruit + Veg. has been square root transformed. Standardized beta coefficients (β) are presented.

* p < 0.05

** *p < 0.01*.

**Table 3:** Summary of multiple linear regression analysis for sedentary time (ST) predicting dietary intake variables in children (8–12 years) (*n* = 136).

	**Dietary fat (% total kcal)**	**Mean daily dietary carbohydrate (% of total kcal)**	**Mean daily dietary protein (% of total kcal)**	**Mean daily glycemic load**	**Mean daily fruits + veg. (servings) per 1,000 kcal**
**Variable**	β	β	β	β	β
Age	−0.058	0.028	0.038	0.048	0.010
Male	−0.004	−0.083	0.161	−0.082	−0.076
Hispanic	0.110	−0.224^*^	0.256^**^	−0.220^*^	−0.097
Annual household income (quartiles)	0.077	−0.097	0.067	−0.076	−0.010
BMI percentile	0.100	−0.158	0.141	−0.199^*^	0.125
ST	0.294^**^	−0.282^**^	0.078	−0.242^**^	−0.076

ST, average daily minutes sedentary time (per hour of valid wear time) on valid days measured by waist-worn accelerometer. kcal, kilocalories. BMI, Body Mass Index. Dietary intake variable measured by multiple 24-h recalls. Fruit + Veg. has been square root transformed. Standardized beta coefficients (β) are presented.

* p < 0.05

** *p < 0.01*.

## Discussion

This study makes an important contribution to the literature as one of the first studies to combine an objective monitor-based activity measure with multiple 24-h diet recalls to examine the associations of MVPA and ST with specific components of dietary intake in children. Results indicating that greater ST is associated with a greater proportion of energy intake from dietary fat are consistent with a number of previous studies (25–29). The largest contributors to fat intake in the current study were lunchmeats, chicken nuggets, fast food meals, oils, butter, chips, and ice cream. Children may consume these types of high fat foods and snacks while watching TV or engaging in other screen behaviors (30, 31) as these activities may distract normal satiation cues (32–34) or trigger hunger cues through food advertising (35, 36). The observed association between higher MVPA and a lower proportion of energy intake from dietary fat has appeared less frequently in the literature (37). However, the majority of prior studies in this area use either parent- or self- report measures of children's sedentary or screen time and physical activity, which may be prone to recall or reporting biases that obscure relationships (10). Previous studies also typically measure dietary intake through food frequency measures, which suffer from other methodological weakness such as underestimation and lower reliability compared to the 24-h dietary recall (38).

A novel finding of this study was that greater MVPA and less ST were associated with a greater proportion of total dietary energy intake from carbohydrates particularly foods with higher glycemic load (i.e., simple sugars, white bread, white rice). Children may consume a greater proportion of energy intake from carbohydrates such as granola bars, sweetened cereals, pastry, candy, pretzels, bread, and sports drinks before, during, or after organized sports and at activity-based classes/lessons (e.g., karate, dance). This may be because these foods dominate the concessions sold at these events, are often given out as postgame treats, and serve a substitute for regular meals that are disrupted due to sports games and practices (39–41). In fact, a growing body of evidence suggests that youth sports may expose children to an unhealthy food environment (42, 43). Another potential explanation is that increased physical activity is a behavioral response to carbohydrate intake (44, 45). Consuming foods with a high glycemic index may reduce feelings of fatigue or replenish skeletal muscle energy, which can induce physical activity behaviors (46). Also, the body may increase musculoskeletal activity in a compensatory manner to reduce elevated blood glucose levels in response to high glycemic foods (47–49).

This study had some limitations. Due to the cross-sectional design, inferences about directionality of causal effects cannot be made. Additionally, many of the observed effects sizes are small. Although using 24-h dietary recall methodology in children offers improvements over parent proxy-report and food frequency instruments, there are more objective measures of dietary such as the use of urine metabolites doubly labeled water, and food photography that have improved reliability and validity. Also, approximately a quarter of potential participants did not have complete 24-h dietary recall data, however, these rates of non-response and non-compliance they are comparable to other studies using this methodology in children (50, 51). Furthermore, although the accelerometer provided objective measures of MVPA and ST, amounts may be underestimated due to accelerometer non-wear. For improved accelerometer wear time compliance, a wrist-worn model may be used. Additionally, including children with < 4 valid days of accelerometer wear may reduce the reliability of these data and the ability to detect significant associations. Also, results may not be generalizable to children younger than age 8 or adolescents.

## Conclusions

Overall, this project addressed important methodological limitations of prior work in being one of the first studies to use an objective activity monitor combined with multiple child-report 24-h dietary recalls in children. Despite its direct health benefits, physical activity may be associated with consuming a greater proportion of total energy intake from carbohydrates, especially those with a higher glycemic index such as simple sugars, white bread, white rice. These results underscore that diet, physical activity, and sedentary behaviors might not cluster together in a manner that optimizes health outcomes. Further research is needed to understand the differential implications of these unique behavioral interrelations for diabetes, cardiovascular, and obesity risk.

## Author contributions

GD and SS formulated the research questions. GD designed the study. SO processed and cleaned the data. GD analyzed the data and wrote the article. SO, BB, JM, and SS reviewed and edited the article.

### Conflict of interest statement

GD and JM have received consulting payments from the Dairy Council of California. GD has received travel funding from the National Physical Activity Plan Alliance. GD has received consulting payments from the National Collaborative on Childhood Obesity Research. These organizations had no role in the design, conduct of the study, collection, analysis/interpretation of the data, or preparation, review, approval of the manuscript. The remaining authors declare that the research was conducted in the absence of any commercial or financial relationships that could be construed as a potential conflict of interest.
